# Asthma co-morbidities in Nigerian children: prevalence, risk factors and association with disease severity and symptoms control

**DOI:** 10.11604/pamj.2020.35.91.18470

**Published:** 2020-03-26

**Authors:** Bankole Peter Kuti

**Affiliations:** 1 Department of Paediatrics and Child Health, Obafemi Awolowo University, Ile-Ife, Nigeria

**Keywords:** Allergic rhino-conjunctivitis, childhood asthma, co-morbidities, symptoms control

## Abstract

**Introduction:**

Prompt recognition and management of co-morbidities is an important step in ensuring optimal childhood asthma symptoms control. This study sets out to determine the prevalence, predictive factors and association of co-morbidities with asthma severity, lung functions and symptoms control in Nigerian children.

**Methods:**

Children (aged 2 to 15 years) with physician-diagnosed asthma at the Wesley Guild Hospital, Nigeria were consecutively recruited. Asthma co-morbidities, severity and levels of symptoms control were assessed using standard definitions. Lung functions of children ≥ 6 years were also measured. Factors predictive of asthma co-morbidities and association of co-morbid conditions with asthma severity, lung functions and symptoms control were determined using univariate and multivariate analyses.

**Results:**

A total of 186 children (male: female 1.4:1) were recruited and the majority (81.0%) had mild intermittent asthma. Forty (21.5%) had suboptimal symptoms control and 112 (60.2%) had associated co-morbidities. Allergic rhinitis and/or conjunctivitis (41.4%) were the most common co-morbidities. Predictors of concomitant presence of allergic rhinitis among the children were older age group ≥ 6 years (OR = 2.488; 95%CI 1.250-4.954; p = 0.036) and lack of exclusive breastfeeding (OR = 2.688; 95%CI 1.199 -5.872; p = 0.020) while obesity/overweight (OR = 6.300; 95%CI 2.040-8.520; p = 0.003) and Allergic rhinitis (OR = 2.414; 95%CI 1.188-6.996; p = 0.049) were determinants of persistent asthma. Suboptimal symptoms control was associated with having concomitant allergic rhinitis (p = 0.018), however no comorbid condition predicted lung function impairment.

**Conclusion:**

About two-thirds of children with asthma had co-morbidities and allergic rhino-conjunctivitis was the most common. School age group and early introduction to breast milk substitutes predict the presence of these co-morbidities which also affect asthma severity and control.

## Introduction

Childhood asthma is a leading cause of chronic respiratory disease in children [1]. It is a major cause of ill health, hospitalisation and emergency room visits as well as school absenteeism in children [1, 2]. Childhood asthma adversely affects the quality of life of children and that of their parents/caregivers [3]. Asthma is responsible for 15 percent of disability adjusted life years and over 334 million persons were recently estimated to be affected with the disease [1]. The major goal of childhood asthma management is to achieve optimal symptoms control which will enable children with asthma to live a normal life and achieve their potentials [1]. As desirable as this management goal is, it is often not easily achievable. One of the reasons for poor or suboptimal symptoms control in childhood asthma is poor recognition and or management of asthma co-morbidities [1, 4, 5]. These co-morbidities were reported to affect the severity and manifestations of the disease as well as the level of symptoms control [1, 4-8]. Asthma co-morbidities also increase the cost of management of childhood asthma and they may mimic or override asthma symptoms in children, thereby leading to over diagnosis or underdiagnosis of the disease [5-8]. Co-morbidities in childhood asthma include allergic diseases like rhinitis, conjunctivitis and dermatitis [1]. Others include conditions affecting the respiratory system like nasal polyposis, adenotonsillar hypertrophy, sleep breathing disorders and chest infections [1]. Non-respiratory diseases like gastroesophageal reflux diseases (GERD), childhood obesity, hyperlipidaemia, diabetes mellitus, chronic obstructive pulmonary diseases (COPD) and psychological/emotional disturbances have also been reported to be higher in children with asthma than their peers without asthma [6-8].

Various studies from developed countries have reported allergic rhinitis as a leading co-morbid condition in childhood asthma observed in 60 to 80% of these children as against up to 30 to 40% of non-asthmatics [6-8]. Similarity in the pathophysiology of asthma and allergic rhinitis was proposed as one of the reasons for the increased prevalence of allergic rhinitis in children with asthma [9]. The prevalence of GERD in children with asthma was reported to range from 19.3% to 65% [10]. There is a cause and effect relationship between asthma and GERD as asthma medications can increase GERD, which in turn can provoke asthma symptoms [10]. Childhood obesity often reported as an important co-morbidity of asthma also has a cause and effect relationship with asthma [11]. Poorly controlled asthma symptoms; high dose steroid use and exercise intolerance may result in poor sleep, depression and obesity [11]. Likewise these conditions may affect compliance with medications, symptoms perception and severity of asthma [11, 12]. These will ultimately affect symptoms control and quality of life of children with asthma [12]. Unfortunately co-morbidities in childhood asthma are often neglected, poorly addressed and under-reported particularly in Africa and other developing parts of the world where there are bodies of evidence to suggest increasing prevalence of childhood asthma [4, 5, 13]. This poor recognition and management of asthma co-morbidities often results in poor childhood asthma management outcome [4-8]. This study therefore sets out to determine the prevalence and pattern of co-morbidities in children with asthma attending the paediatric chest clinic of a tertiary health facility in southwest Nigeria; to assess the predictive factors associated with these co-morbidities and association of asthma co-morbidities with asthma severity, lung function impairments and symptoms control.

## Methods

**Study design:** this was a hospital-based cross-sectional study.

**Study location:** this study was carried out at the paediatric chest clinic of the Wesley Guild Hospital (WGH), Ilesa, south west Nigeria over an 18-month period (May 201^7^ to October 201^8^). The clinic attends to children with respiratory conditions predominant of which is bronchial asthma. The WGH is a tertiary arm of the Obafemi Awolowo University Teaching Hospitals Complex (OAUTHC) which provides general and specialised care for children in the south west part of Nigeria.

**Sample size determination:** the minimum sample size for this study was estimated using open Epi sample size software^(R)^ [14] and adjusted for a finite population based on the 300 registered asthmatics at the paediatric chest clinic. Assumptions made included 33.3% prevalence of allergic rhinitis in children with asthma, [15] alpha value of 5% at 95% Confidence interval (CI) and power of 80. A minimum sample size of 180 was calculated.

**Ethical consideration:** ethical approval for this study was obtained from the Ethics and Research Committee of the OAUTHC, Ile- Ife. Informed consent and assent were obtained from the caregivers and children > 6years respectively.

**Study procedure:** all children (aged 2 to 15 years) with asthma who presented for routine clinic follow up during the study period were consecutively recruited. For this study, Asthma was diagnosed as history of recurrent episodes of cough, wheezing, chest tightness, and shortness of breath which resolves spontaneously or with the use of bronchodilators [1]. For children ≥ six years, the diagnosis of asthma was further confirmed by demonstrating significant improvement in lung function (forced expiratory volume in one second (FEV1) ≥ 12%) following the use of short acting bronchodilator [1]. Information obtained from the caregivers and the study participants included the age (in years) and sex of the children. Parental occupation and highest level of education was also obtained which was used to derive the socio-economic class of the study participants using the method validated by Ogunlesi [16]. The age at diagnosis of asthma, family history of asthma and other allergic diseases were also obtained. Also of interest in this study was early nutritional history of the study participants including history and duration of breastfeeding and use of breast milk substitute. The presence of household pets and poultry were also noted, likewise the type of household fuel used for cooking, heating and lighting. Households where electric and cooking gas were used for cooking were classified as using “clean fuel,” while the use of biomass fuels of any kind, kerosene stove and coal were classified as unclean fuel” [17]. The numbers of individuals that share the same bed room in the household of the study participants were also noted and overcrowded household was defined as having three or more individuals sharing a standard bedroom with the child [18]. All the study participants were thoroughly examined specifically for features of allergy and other asthma co-morbidities. For the purpose of this study, asthma co-morbidities were defined as respiratory and non-respiratory conditions in children with asthma that can affect the outcome of asthma management [8]. Allergic rhinitis was defined based on the presence of recurrent nasal itching, discharge, sneezing and nasal obstruction induced by exposure to allergens according to Allergic Rhinitis and its Impact on Asthma (ARIA) criteria [19]. Allergic dermatitis was defined based on the presence of pruritic vesicular, weeping or crusting eruptions. These lesions may also be dry, scaly, and lichenified found mostly in large joint flexures according to Williams *et al.* [20]. Children with associated itchy eyes with brownish discoloration of sclera and other eye symptoms were referred to the ophthalmologist for the diagnosis and management of allergic conjunctivitis. GERD in these children was diagnosed based on a score of ≥ 8 on the validated GerdQ self-assessment questionnaire [21]. Anthropometric parameters (weight in kilogrammes, height in metres and Body mass Index (BMI) in kilogrammes per square metre) of the children were taken using standard methods. Obesity was defined as BMI Z score > +2 SD; overweight was defined as BMI Z score > + 1 SD while wasting was defined as BMI Z score < -2 SD on the WHO growth reference chart [22].

**Lung function assessment:** children ≥ 6 years had their lung function assessed using a standard spirometer (MIR Spirolab III, Medical International Research srl Italy) following ATS/ERS guidelines. Parameter of interest included FEV1, Forced vital capacity (FVC), FEV1/FVC and peaked expiratory flow rate (PEFR). The children with obstructive, restrictive or mixed ventilatory pattern were classified as having impaired lung function [23]. The severity of asthma in the study participants at presentation was categorised into intermittent, mild, moderate and severe persistent asthma based on the Expert Panel report of the National Asthma Education and Preventive Programme (NAEPP) guidelines [24] while symptoms control was assessed using GINA guidelines into well controlled, partly controlled and uncontrolled symptoms [1]. For this study, children with uncontrolled and partly controlled symptoms were further categorised into suboptimal symptoms control, while well controlled symptoms were classified as optimal symptoms control.

**Data analysis:** this was done using Statistical Program for Social Sciences (SPSS) software Version 17.0 (SPSS Inc, Chicago 2008). The number of children with co-morbidities was expressed as a percentage of the total number of study participants to obtain the prevalence of asthma co-morbidities. Other categorical variables were expressed as proportions and percentages. The ages, weight, height, lung function parameters and other continuous variables were tested for normality and summarized using mean and standard deviations (SD) or median and interquartile range (IQR) as appropriate. Differences between the means (SD) of continuous variables were analysed using Student's t-test, whereas categorical variables were analysed using Pearson's Chi-square test and Fisher's exact test, as appropriate. Variables that were significantly associated with the outcomes using univariate analysis were entered into binary logistic regression model to determine the independent predictors of dichotomised outcomes (presence vs. absence of allergic rhinitis; intermittent vs. persistent asthma). Results were interpreted as Odd ratio (OR) and level of significance at 95% confidence interval (CI) was taken as p < 0.05.

## Results

Socio-demographic characteristics of the children are highlighted in Table 1. The ages of the children ranged from 2 to 15 years with a median (IQR) age of 7.4 (3.8-10.3) years. The majority (48.3%) of the study participants were school age children and only 25 (13.4%) were teenagers. There were 108 (58.1%) males with a male: female of 1.4:1. Age at diagnosis of asthma: This ranged from 10 month to 14 years with a median (IQR) of 4.0 (2.0-7.1) years. About 40.0% of the children were diagnosed at school age. Socio-economic classes of the study participants: The majority (90.8%) of the children were from upper and middle classes, only 19 (8.6%) were from low socio-economic class Duration of exclusive breastfeeding: This ranged from 0 to 16 months with a mean (SD) duration of 4.9 (1.9) months. One hundred and two (54.8%) of the study participants were exclusively breastfed for the first six month of life. Number of children in the household: The mean (SD) children per household was 2.9 (1.4) which ranged from 1 to 9 children per household. Only 35 (18.8%) lived in overcrowded homes. Household fuel: About one-third (34.4%) of the study participants used clean fuel for household cooking and lighting. The rest (65.6%) use unclean fuel which comprises kerosene (59.2%), firewood (4.9%) and coal (2.5%). Family history of asthma and allergic diseases: positive family history of asthma was obtained in 98 (52.7%) of the study participants. These family relations included parents (35.9%); siblings (7.1%); grandparents (6.0%) and uncle/aunt (4.9%). Severity of asthma at presentation: majority (81.8%) of the children had intermittent asthma, while others (18.2%) had persistent asthma which included mild (12.4%), moderate (4.8%) and severe persistent asthma (1.1%). Level of asthma symptoms control: forty (21.5%) of the children had suboptimal symptoms control which was partly controlled in 35 (18.8%) and uncontrolled in 5 (2.7%) during the study period.

**Table 1:** Socio-demographic and general characteristics of the study participants as related to the presence of asthma co-morbidities

Variables	Asthmatics with co-morbidities n = 112 (%)	Asthmatics without co-morbidities n = 74 (%)	Total 186	x^2^	p-values
**Sex**					
Male	67 (59.8)	41 (55.4)	108	0.355	0.551
Female	45 (40.2)	33 (44.6)	78		
**Age-range (years)**					
≤5	30 (26.8)	41 (55.4)	71	14.439	**0.001**
6-12	64 (57.1)	26 (35.1)	90		
≥12	18 (16.1)	7 (9.5)	25		
**Period diagnosis of asthma made**					
Infancy	10 (8.9)	10 (13.5)	20	8.564	**0.036**
Toddler	33 (29.5)	32 (43.2)	65		
Preschool	15 (13.4)	12 (16.2)	27		
School age	54 (48.2)	20 (27.0)	74		
					
Exclusively breastfed	60 (53.6)	42 (56.8)	102	0.131	0.717
Not exclusively breastfed	51 (46.4)	32 (43.2)	83		
					
**Socio-economic class**					
Upper	49 (43.8)	36 (48.6)	85	0.539	0.764
Middle	53 (47.3)	31 (41.9)	84		
Low	10 (8.9)	7 (9.5)	17		
**Cooking fuel**					
Clean fuel	40 (35.7)	24 (32.4)	64	0.213	0.645
Unclean fuel	72 (64.3)	50 (67.6)	122		
**Overcrowding**					
Yes	21 (18.8)	14 (18.9)	35	0.001	0.977
No	91 (81.2)	60 (81.1)	148		
**Family history of asthma/allergic diseases**					
Yes	61 (54.5)	37 (50.0)	98	0.323	0.570
No	51 (45.5)	37 (50.0)	88		
**Severity of asthma**					
Intermittent	89 (79.5)	63 (85.6)	152	0.959	0.327
Persistent	23 (20.6)	11 (14.4)	34		

*The figures in parentheses are percentages along the columns

**Examination findings:** anthropometric parameters: the weight of the study participants ranged from 10.0 to 63.0 kg with a mean (SD) of 25.2 (11.7) kg. The median (IQR) height was 1.2 (0.9-1.4) m, which ranged from 0.8 to 1.7m. The BMI ranged from 9.8 to 25.4kg/m^2^ with a mean (SD) of 16.1 (2.4) kg/m^2^. The majority of the children (89.8%) had normal nutritional status; eight (4.3%) were wasted, seven (3.8%) were overweight and nine (4.8%) were obese. Lung function assessment of the children: This was done in 114 children (≥ 6 years) one child could not perform an acceptable spirometry. The Mean (SD) values of the FEV_1_, FVC and FEV_1_/FVC of the study participants were 1.6 (0.6) L; 1.9 (0.7) L and 90.1 (18.1)% respectively. Seventy-four (64.9%) of the 114 children had normal ventilatory pattern while 40 (35.1%) had impaired lung function. These included 29 (30.8%) with obstructive ventilatory pattern and 11 (4.3%) with mixed pattern.

**Asthma co-morbidities among the children:** one hundred and twelve children (60.2%) had one form of asthma co-morbidities or another. Table 2 highlights the asthma co-morbidities as related to age and sex distribution of the children with asthma. Allergic rhinitis (23.7%) with or without conjunctivitis and allergic conjunctivitis alone (17.7%) were the most common co-morbid conditions. Other co-morbidities included obesity/overweight; atopic dermatitis; adenoidal hypertrophy and GERD. While adenoidal hypertrophy was more frequently observed among preschool male children, GERD and obesity/overweight were more common among school age female children (Table 2).

**Table 2:** Asthma co-morbidities as related to the age and sex distribution of the children

*Asthma comorbidities	Age and sex distribution of the children with asthma (in years)							
	≤5 (n = 72)		6 -12 (n = 90)		>12 (n = 24)		*Total	
	Males n = 48	Females n = 24	Males n = 52	Females n = 38	Males n = 8	Females n = 16		
#Allergic rhinitis	5 (10.4)	0 (0.0)	19 (36.5)	12 (31.6)	5 (62.5)	2 (12.5)	44	
Allergic conjunctivitis	5 (10.4)	5 (10.4)	14 (26.9)	6 (15.8)	3 (37.5)	0 (0.0)	33	
Overweight /Obesity	2 (4.2)	1 (2.1)	2 (3.8)	2 (5.3)	1 (12.5)	3 (18.8)	11	
Atopic dermatitis	2 (4.2)	2 (3.9)	3 (5.8)	1 (2.6)	2 (25.0)	0 (0.0)	9	
Wasting	0 (0.0)	1 (1.0)	0 (0.0)	6 (15.8)	0 (0.0)	1 (4.0)	8	
GERD	0 (0.0)	0 (0.0)	0 (0.0)	5 (13.0)	1 (12.5)	2 (8.0)	6	
Nasal polyps	0 (0.0)	0 (0.0)	2 (3.8)	1 (2.6)	2 (25.0)	0 (0.0)	5	
Adenoidal hypertrophy	3 (6.3)	1 (1.9)	1 (1.9)	0 (0.0)	0 (0.0)	0 (0.0)	5	
Sinusitis	0 (0.0)	0 (0.0)	1 (1.9)	1 (2.6)	0 (0.0)	0 (0.0)	2	

* Some children had more than one co-morbid conditions; # with or without conjunctivitis

**Factors associated with asthma co-morbidities among the children:** increasing age range (≥ 6 years) was significantly associated with having asthma co-morbidities among the study participants. Likewise advancing age at diagnosis of asthma was significantly associated with the presence of concomitant co-morbidities (Table 1) Socio-economic classes; household characteristics and type of household cooking fuels were not associated with the presence of co-morbidities of asthma.

**Risk factors for the presence of allergic rhinitis among the children:** Table 3 highlights the risk factors associated with the presence of allergic rhinitis among the children with asthma. Advancing age (≥ 6 years) (χ^2^ = 14.439; p = 0.001) and lack of exclusive breastfeeding (33.3% vs. 15.7%; χ^2^ = 7.277; p = 0.007) were significant risk factors to developing allergic rhinitis among the children.

**Table 3:** Risk factors for the presence of allergic rhinitis among the children with asthma

Variables	Asthmatics with Allergic rhinitis n = 44 (%)	Asthmatics without Allergic rhinitis n = 142 (%)	Total 186	x^2^	p-values
**Sex**					
Male	26 (59.1)	82 (57.5)	108	0.025	0.895
Female	18 (40.9)	60 (42.5)	78		
**Age-range at presentation (years)**					
≤5	5 (11.4)	67 (47.2)	72	17.892	<0.001
6-12	32 (72.7)	58 (40.8)	90		
≥12	7 (15.9)	17 (12.0)	24		
**Period diagnosis of asthma made**					
Infancy	4 (9.1)	16 (11.3)	20	11.077	0.011
Toddler	8 (18.2)	57 (40.1)	65		
Preschool	5 (11.4)	22 (15.5)	27		
School age	27 (61.3)	47 (33.1)	74		
Exclusively breastfed	16 (36.4)	86 (60.6)	102	7.277	0.007
Not exclusively breastfed	28 (73.6)	56 (39.4)	84		
**Socio-economic class**					
Upper	20 (45.5)	65 (45.8)	85	0.209	0.905
Middle	20 (45.4)	64 (45.1)	84		
Low	4 (9.1)	13 (9.1)	17		
**Cooking fuel**					
Clean fuel	18 (40.9)	46 (32.4)	64	1.079	0.299
Unclean fuel	26 (59.1)	96 (61.6)	122		
**Overcrowding**					
Yes	9 (20.5)	26 (18.3)	35	0.101	0.750
No	35 (79.5)	116 (81.7)	151		
**Family history of asthma/allergic diseases**					
Yes	26 (50.1)	72 (50.7)	98	0.948	0.330
No	18 (49.9)	70 (49.3)	88		

*The figures in parentheses are percentages along each column

**Asthma co-morbid conditions as related to asthma severity:** Table 4 highlights the association of asthma severity at presentation, lung function assessment and level of symptoms control as related to the various asthma co-morbidities Children with allergic rhinitis were more likely to have persistent asthma as 16 (39.0%) of the 41 children with persistent asthma had allergic rhinitis compared to 24 (14.1%) of the 170 children without allergic rhinitis (p = 0.004). Likewise the presence of obesity/overweight, atopic dermatitis and adenoidal hypertrophy were significantly associated with more severe asthma symptoms among the children.

**Table 4:** Asthma co-morbidities as related to severity, lung function impairment and level of symptoms control among the study participants

Asthma co-morbidities	Asthma severity		
	Intermittent n = 152 (%)	Persistent n = 34 (%)	p-value
Allergic rhinitis	31 (20.4)	13 (38.2)	0.049
Allergic conjunctivitis	24 (15.8)	9 (26.5)	0.142
Overweight /Obesity	5 (3.3)	6 (17.6)	0.001
Atopic dermatitis	6 (3.9)	3 (8.8)	0.267^#^
Wasting	7 (4.6)	1 (2.9)	0.651^#^
GERD	5 (2.8)	1 (2.9)	0.917^#^
Adenoidal hypertrophy	1 (0.7)	4 (11.8)	<0.001^#^
Nasal polyps	3 (2.1)	2 (5.8)	0.250^#^
Sinusitis	1 (0.7)	1 (2.9)	0.309^#^
**Asthma co-morbidities**	**Lung functions assessment^**		
	**Normal lung functions n = 74 (%)**	**Impaired lung functions n = 40 (%)**	
Allergic rhinitis	21 (28.4)	17 (42.5)	0.140
Allergic conjunctivitis	20 (27.0)	13 (32.5)	0.539
Overweight /Obesity	7 (9.5)	2 (5.0)	0.383^#^
Atopic dermatitis	2 (2.7)	3 (7.5)	0.246^#^
Wasting	3 (4.1)	4 (10.0)	0.219^#^
GERD	5 (6.8)	1 (2.5)	0.331^#^
Adenoidal hypertrophy	1 (1.4)	0 (0.0)	0.354^#^
Nasal polyps	3 (4.1)	2 (5.0)	0.246^#^
Sinusitis	0 (0.0)	1 (2.5)	0.354^#^
	**Level of asthma symptoms control**		
**Asthma co-morbidities**	**Optimal symptoms control n = 146 (%)**	**Sub-optimal control n = 40 (%)**	**p-value**
Allergic rhinitis	28 (19.2)	15 (37.5)	0.018
Allergic conjunctivitis	25 (17.1)	8 (7.5)	0.689
Overweight /Obesity	9 (6.2)	2 (5.0)	0.778^#^
Atopic dermatitis	7 (4.8)	2 (5.0)	0.957^#^
Wasting	5 (3.4)	3 (7.5)	0.260^#^
GERD	5 (3.4)	1 (2.5)	0.763^#^
Adenoidal hypertrophy	3 (2.1)	2 (5.0)	0.343^#^
Nasal polyps	4 (2.7)	1 (2.5)	0.933^#^
Sinusitis	0 (0.0)	1 (2.5)	0.079^#^

The figures in parentheses are percentages along each column

^ Lung function assessment was achieved in 114 study participants.

# Fischer’s

exact test applied

**Asthma co-morbidities as related to lung function impairment among the children:** none of the asthma co-morbidities were significantly associated with impaired lung function among the study participants.

**Asthma co-morbidities and symptoms control:** only the presence of concomitant allergic rhinitis was significantly associated with suboptimal asthma symptoms control (Table 4).

Predictors of the presence of asthma co-morbidity (allergic rhinitis) and persistent asthma in the study participants using binary logistic regression analysis: Table 5 highlights the binary regression analysis to determine the predictors of the presence of allergic rhinitis and persistent asthma among the children with asthma The variables found to be significantly associated with the presence of allergic rhinitis and persistent asthma (dependent variables) were fitted into regression analysis model to determine the independent predictors of the outcomes. Older age group (≥ 6 years) (OR = 2.488; 95%CI 1.250-4.954; p=0.036) and lack of exclusive breastfeeding (OR = 2.688; 95%CI 1.199 - 5.872; p = 0.020) were predictors of concomitant presence of allergic rhinitis among the children with asthma; while Allergic rhinitis (OR=2.414; 95% CI 1.188-6.996; p = 0.049), obesity/overweight (OR = 6.300; 95%CI 2.040-8.520; p = 0.003) were determinants of persistent asthma.

**Table 5:** Binary logistic regression analysis to determine the independent predictors of allergic rhinitis and persistent asthma

	Predictors of concomitant presence of allergic rhinitis				
Variables	Coefficient of regression	Standard error	p-value	Odd ratio	95%CI of Odd ratio Lower - Upper
Age range at presentation	0.717	0.342	0.036	2.488	1.250 – 4.954
Age range at asthma diagnosis	0.144	0.217	0.506	0.865	0.564 – 1.326
Lack of exclusive breastfeeding	0.875	0.376	0.020	2.688	1.199 – 5.872
	**Predictors of persistent asthma**				
Allergic rhinitis	0.838	0.426	0.049	2.416	1.188 – 6.996
Overweight/obesity	1.932	0.652	0.003	6.300	2.040 - 8.520
Adenoidal hypertrophy	3.646	1.989	0.083	20.133	0.635 – 35.657

CI Confidence interval

## Discussion

This study highlights a high prevalence of comorbid conditions among Nigerian children with asthma attending a specialist clinic in a tertiary health facility. It also highlights the predictive factors for these comorbidities and their association with asthma severity, lung function impairment and symptoms control. About two-thirds of the study participants had recognisable asthma co-morbidities. This finding was corroborated by reports of high prevalence of asthma co-morbidities among children with asthma in developed and developing countries [6-8, 15, 25]. In agreement with other reported studies, the most common asthma co-morbidities were allergic diseases like allergic rhinoconjunctivitis and atopic dermatitis [6-8, 15, 25]. Allergic disorders may co-exist with asthma as a part of the so called “atopic march” - a phenomenon which describes the progression of one form of allergic disease to another [26]. Atopic dermatitis from super antigen sensitisation progressing to asthma and then to allergic rhinitis [27]. The United airway concept also explains the high prevalence of allergic rhinitis in children with asthma [9]. As the upper and part of the lower airways are lined by the same epithelium, they tend to react to same stimuli (Nasobronchial reflex) and share similar pathologies including allergic diseases [9]. As observed in this study and similar reports [8, 26, 28], the presence of allergic rhinitis in children with asthma was associated with suboptimal asthma symptoms control. Kang *et al.* [29] reported that children with asthma and concomitant allergic rhinitis have more frequent asthma exacerbations leading to more emergency room visits and hospitalisation than asthmatics without allergic rhinitis. This implies that in children with asthma, active search for co-morbidities particularly allergic disorders are of paramount importance for a more holistic asthma care delivery.

Lack of exclusive breastfeeding and subsequent early introduction to breast milk substitutes (BMS) was an independent determinant of asthma comorbidities particularly allergic rhinitis in this study. Breast milk has been recommended as the most ideal food for optimal infant nutrition [30]. Apart from meeting the nutritional requirements of infants especially in early infancy, it has also been reported to reduce the incidence of allergic diseases in children [31]. The International Study of Asthma and Allergic diseases in children (ISAAC) phase two study clearly demonstrated that breastfeeding was associated with less wheezing disorders and allergic diseases in low income countries [13]. In a systematic review and meta-analysis of prospective studies on effects of breastfeeding on atopic diseases and asthma, Oddy and Peat [32] reported strong protective effects of breastfeeding against atopic diseases. Breast milk protein is less immunogenic and allergogenic and hence not likely to stimulate food allergic and other allergic diseases in children unlike non-human proteins in BMS which may stimulate immune reaction leading to allergic diseases [31]. Counseling mother on the need for exclusive breastfeeding in early infancy is therefore an important way of reducing the burden of childhood asthma and other allergic diseases. Exploring the relationship between the presence of co-morbidities and severity of childhood asthma revealed that children with asthma and concomitant allergic rhinitis, obesity and Adenotonsillar hypertrophy had more severe forms than those without these co-morbidities and are more likely to require controller medications (inhaled corticosteroid) for asthma control. In a review of the influence of comorbid conditions on asthma, Boulet [7] stated that comorbid conditions can change asthma phenotype, they can be a part of the same pathological process, they can result from the same or similar environmental exposures or acts as confounders in the diagnosis and assessment of asthma [7].

Allergic rhinitis was found in this study to be a predictor of persistent asthma among the children with asthma, and was significantly associated with suboptimal symptoms control. Though the level of significance was marginal in this study, these findings were corroborated by other workers [26, 28]. Apart from the “united airway” concept that suggests that upper and lower airway inflammation are of similar type, rhinitis has also been reported to facilitate increased production of bone marrow progenitors and the release of inflammatory mediators into the circulation which eventually affect the lower airway [6-9]. Additionally, with allergic rhinitis, there is increased mouth breathing as a result of nasal blockage. This leads to poor humidification and filtration of inspired air increasing lower airway exposure to airborne allergens, [9] hence the increased asthma severity and suboptimal symptoms control seen in asthmatic children with concomitant allergic rhinitis. Childhood obesity in this study was an independent predictor of asthma severity (persistent asthma). Overweight and obese children with asthma were observed in this study to be 6 times more likely to have persistent asthma than non-obese non-overweight asthmatics. The relationship between asthma severity and childhood obesity was also reported by other workers [11, 12]. Quinto *et al.* [12] suggested that children with obesity have more severe forms of asthma, reduced response to asthma medications and suboptimal symptoms control [12]. Systemic inflammatory process from adipocytokines and a reduced response to asthma medications are possible mechanisms of the relationship between childhood asthma and obesity [11, 12]. Eneli *et al.* [33] in a systematic review and meta-analysis of 15 published works on effects of weight reduction on asthma symptoms control and other asthma related outcomes reported that weight reduction improved asthma related outcomes including symptoms control and response to medications [33]. Weight reduction programme should therefore be an integral part of asthma management plan of children with asthma and obesity.

Adenoidal hypertrophy in this study was weakly associated with persistent and severe asthma. Though there is paucity of reports on the impact of concomitant adenotonsillar hypertrophy on severity of childhood asthma, however its association with increasing prevalence of asthma had been variously reported [34, 35]. Adenotonsillar hypertrophy is the major cause of obstructive sleep apnoea (OSA) and sleep related breathing disorders in children. Sleep breathing disorders were reported as not only being common in children with asthma than non-asthmatics, but also increase the burden of childhood asthma and impair the quality of life of asthmatics [34, 35]. OSA is associated with increased airway collapse and the systemic inflammation that often accompanies it may also affect asthma control. Evidence of bronchial neutrophilia and markers of airway inflammation have been reported in children with OSA even without asthma [35]. Treatment of OSA in children with asthma was observed to improve their asthma-related quality of life [34, 35]. Lung function impairments was observed in about one-third of the children studied which agrees with reports by Bacharier *et al.* [36] among American children with asthma. However, the presence of asthma co-morbidities was not associated with lung function impairment among the study participants. This is at variant with reported studies, (3637) Carsley [37] for instance reported that asthma co-morbidities were associated with long term lung impairment among Dutch children with asthma; Weiss *et al*. [38] also reported that there is an average of 5% reduction in FEV_1_ among female American asthmatics over a period of 3 years. This connotes that longitudinal monitoring of lung function rather than crossectional survey will be more appropriate to determine the role of asthma and its co-morbidities on lung functions of children. Prospective longitudinal studies particularly in African children to highlight the effects of asthma co-morbidities on lung function will be worthwhile. This study is limited by non-assessment of psychological and emotional aspects of asthma co-morbidities as related to asthma severity and symptoms control. Also the inability to objectively diagnose the presence of OSA due to non-availability of polysomnography is a limitation of this study. Nevertheless, the study has highlighted the burden and predictors of childhood asthma co-morbidities and the effects of these co-morbidities on asthma severity and symptoms control in Nigerian children.

## Conclusion

Asthma comorbidities are very common in Nigerian children with asthma and they are significantly associated with asthma severity and level of symptoms control particularly concomitant allergic rhinitis and obesity. Early exposure to BMS predicts the presence of some of allergic rhinitis among the study participants. Clinicians managing African children with asthma should actively assess them for co-morbid conditions and promptly manage/address these co-morbidities to ensure optimal asthma symptoms.

### What is known about this topic

- Children with asthma often have co-morbid conditions;
- Comorbidities affects asthma manifestations and severity;
- Unrecognized asthma co-morbidities can affect symptoms control.

### What this study adds

- Allergic rhinoconjunctivitis is the most common co-morbid condition in Nigerian children with asthma and it affects asthma severity and symptoms control;
- Older age group (≥ 6 years) and lack of exclusive breastfeeding (early introduction to breast milk substitutes) are independent predictors of asthmatics having concomitant allergic rhinitis;
- Obesity and concomitant allergic rhinitis are determinants of tendencies to require controller medications (persistent asthma) for asthma symptoms control.

## Competing interests

The author declares no competing interests.
